# Brown-like adipose progenitors derived from human induced pluripotent stem cells: Identification of critical pathways governing their adipogenic capacity

**DOI:** 10.1038/srep32490

**Published:** 2016-08-31

**Authors:** Anne-Laure Hafner, Julian Contet, Christophe Ravaud, Xi Yao, Phi Villageois, Kran Suknuntha, Karima Annab, Pascal Peraldi, Bernard Binetruy, Igor I. Slukvin, Annie Ladoux, Christian Dani

**Affiliations:** 1 Université Côte d’Azur, CNRS, Inserm, iBV, Nice, France; 2 Department of Pathology and Laboratory Medicine, University of Wisconsin, Madison, WI 53715, USA; 3 Inserm U910, Faculty of Medicine La Timone, Marseille, France

## Abstract

Human induced pluripotent stem cells (hiPSCs) show great promise for obesity treatment as they represent an unlimited source of brown/brite adipose progenitors (BAPs). However, hiPSC-BAPs display a low adipogenic capacity compared to adult-BAPs when maintained in a traditional adipogenic cocktail. The reasons of this feature are unknown and hamper their use both in cell-based therapy and basic research. Here we show that treatment with TGFβ pathway inhibitor SB431542 together with ascorbic acid and EGF were required to promote hiPSCs-BAP differentiation at a level similar to adult-BAP differentiation. hiPSC-BAPs expressed the molecular identity of adult-UCP1 expressing cells (*PAX3, CIDEA, DIO2)* with both brown (*ZIC1*) and brite (*CD137*) adipocyte markers. Altogether, these data highlighted the critical role of TGFβ pathway in switching off hiPSC-brown adipogenesis and revealed novel factors to unlock their differentiation. As hiPSC-BAPs display similarities with adult-BAPs, it opens new opportunities to develop alternative strategies to counteract obesity.

In mammals, three types of adipocytes coexist, i.e. brown, brite and white, which are all involved in energy balance regulation while having opposite functions. White adipose tissue (WAT) is dispersed throughout the body and is mainly involved in energy storage. In contrast to WAT, brown adipose tissue (BAT) is specialized in energy expenditure. Activated BAT consumes metabolic substrate and burns fat to produce heat via the uncoupling protein (UCP)-1. Brite, also named beige, adipocytes were recently described as brown-like adipocytes and represent a third type of adipocytes recruited in WAT^1^. Brown/brite adipocyte recruitment leads to powerful anti-obesity and anti-diabetic effects in mice. It was previously reported that BAT implants were able to restore normoglycemia in diabetic mice and to reduce obesity in Ob/Ob mice^2^,^3^,^4^. Hence, the notion of transplanting brown/brite adipose progenitors (BAPs) in obese patients as a therapeutic approach to counteract obesity and its associated metabolic complications recently emerged. However, BAT represents a minor fraction of adipose tissue in humans and disappears from most areas with age, persisting only around deeper organs^5^. Human BAPs are hard to isolate in this regard. Therefore, a cellular source of BAPs is urgently needed for the clinic and to better know human brown adipocyte ontogenesis.

Induced pluripotent stem cells (hiPSCs) can be differentiated into multiple cell types *in vitro*. Following the pioneer work of Yamanaka’s group on the generation of patient-specific hiPSCs by reprogramming somatic cells^6^, hiPSCs emerged as an unlimited source of autologous BAPs for cell-based therapy of obesity. However, numerous issues have to be investigated before a therapeutic use of hiPSC-BAPs, including their purification and differentiation into functional adipocytes preferentially at a high level without introduction of exogenous adipogenic genes. The comparison of hiPSC-BAPs with adult-BAPs is also a timely question. Interestingly, white adipocytes generated from hiPSCs can maintain their functional properties for several weeks after transplantation into nude mice^7^,^8^. These data revealed that hiPSCs-white adipocytes could potentially be used to correct metabolic parameters in lipodystrophic patients. Nishio *et al*.^9^ developed a procedure to differentiate hiPSCs into functional brown adipocytes at high efficiency using a hematopoietic cocktail. Remarkably, hiPSCs-brown adipocytes were able to improve glucose tolerance after transplantation in mice. In both works, total differentiated hiPSC cell populations, but not purified APs, were transplanted into mice. Indeed, differentiated hiPSC cultures can be enriched with adipocytes, but also contain other cell types that are unsuitable for transplantation, including undifferentiated iPSCs that can form teratomas. This indicates that purification of hiPSC-BAPs with a high adipogenic capacity is a prerequisite for a hiPSCs-based therapeutic approach. Ahfeldt *et al*.^10^ were able to generate pure brown and white APs from hiPSCs that displayed a high adipogenic capacity but only following transduction with adipogenesis master genes. The need to genetically modify hiPSCs-APs to generate adipocytes clearly illustrates the low adipogenic potential of hiPSCs-derived APs. As recently discussed, the bottleneck is the weak adipogenic potential of APs purified from hiPSCs compared to adult adipose tissue-derived APs^11^. This feature was observed by us and others using different approaches to derive mesenchymal cells from hESCs or hiPSCs^12^. Therefore, events required to unlock differentiation of hiPSCs-BAPs have yet to be identified. Here we describe a set of factors capable of governing hiPSC-BAP differentiation, and interestingly, at a level similar to that of BAPs derived from human adult adipose tissue.

## Results

### An endothelium growth medium and TGFβ pathway inhibition were required to switch on hiPSCs-BAP differentiation

**Figure 1:**
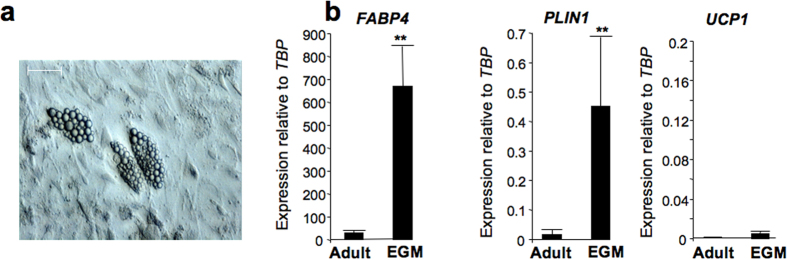
Differentiation of hiPSC-BAPs in EGM adipogenic medium. hiPSC-BAPs were induced to undergo differentiation in a traditional adipogenic medium routinely used for adult APs (Adult) or in the EGM adipogenic medium (EGM). (**a**) Twenty-five days later, multilocular adipocytes were detectable under the microscope only when cells were maintained in the EGM adipogenic medium; bar scale: 50μm. (**b**) RNAs were prepared and analyzed for adipocyte marker expression. Values are means ± SEM. n = 6. *means p < 0.05 and **means p < 0.01.

We recently reported the procedure to selectively generate BAPs and WAPs from hiPSCs depending on the activation of the retinoic acid pathway early during hiPSC differentiation^13^. In agreement with the observation that APs derived from hiPSCs and from hES cells have a low adipogenic potential (for recent review see ref. 11) the screening of traditional adipogenic factors promoting differentiation of adult APs failed to promote formation of hiPSC-BAP adipocytes (Table supplement 1). However, the screening of other types of media showed that the maintenance of hiPSC-BAPs in an endothelium growth medium (EGM) supplemented with traditional adipogenic factors (see Methods) displayed the potential to induce formation of multilocular adipocytes and to enhance expression of adipogenic markers such as *FABP4* and *PLIN1* (Fig. 1). Expression of *UCP1* was very low at the RNA level (Fig. 1b) and undetectable at the protein level (not shown) in these conditions, indicating that factors present in the EGM medium were able to promote BAP differentiation but not at the level of that of adult-BAPs. We hypothesized that anti-adipogenic factors were secreted by hiPSCs-BAPs, thus precluding their differentiation at a higher level.

**Figure 2:**
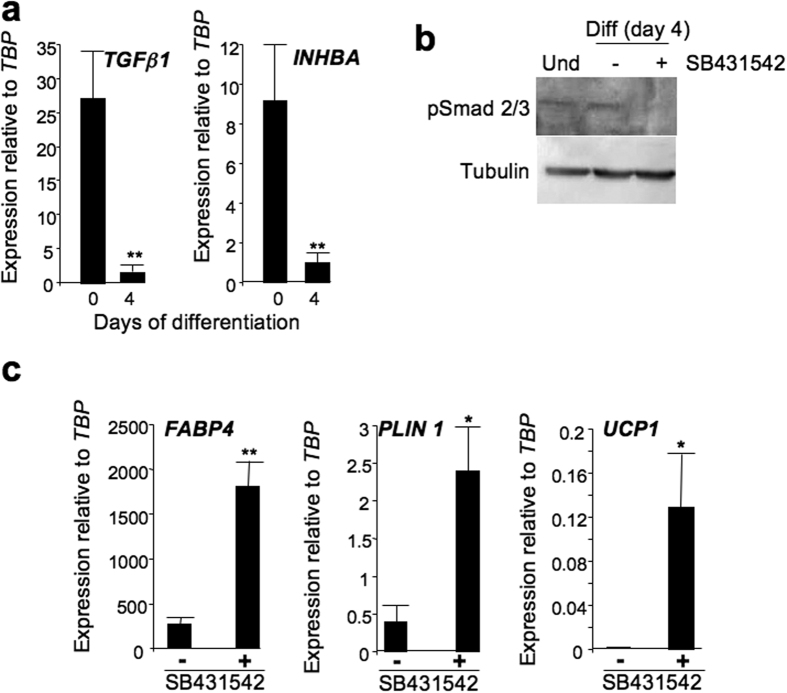
Anti-adipogenic activity secreted by hiPSC-BAPs was reversed by SB431542. (**a**) Expression of TGFβ family members in undifferentiated (day 0) and differentiated hiPSC-BAPs. (**b**) Activated Smad2/3 in undifferentiated and differentiated hiPSC-BAPs in the absence or presence of 5 μM of SB431542. (**c**) hiPSC-BAPs were induced to undergo differentiation in EGM2 adipogenic medium in the absence or presence of 5 μM SB431542. Twenty-five days later, RNAs were prepared and analyzed for the indicated genes. Values are means ± SEM. n = 4. *means p < 0.05 and **means p < 0.01.

The TGFβ pathway recently emerged as a critical anti-adipogenic player through the activation of Smad 2/3^14^,^15^,^16^. The potent anti-adipogenic effect of TGFβ1 was confirmed on adult-BAPs (Fig. S2B). Interestingly, members of the TGFβ family such as *TGFβ1* and *INHBA* were expressed during the first days of hiPSC-BAP differentiation (Fig. 2a). *TGFβ1* and *INHBA* expression was down-regulated in differentiated hiPSC-BAPs compared to the expression levels in undifferentiated cells, but remained at a level sufficient to maintain the Smad2/3 pathway active ((phosphoSmad, Fig. 2b). These data suggested that hiPSC-BAPs secreted bioactive TGFβ family members that might lock hiPSC-BAP differentiation. In agreement with this hypothesis, medium conditioned by hiPSCs-BAPs displayed a potent anti-adipogenic effect on adult-BAP differentiation (Fig. S2). An ERK inhibitor (UO126 at 5 μM) or a p38MAPK inhibitor (SB203580 at 10 μM) was unable to reverse the anti-adipogenic effect of hiPSCs-BAP conditioned medium on adult-BAPs (not shown). In contrast, the anti-adipogenic effect of the conditioned-medium was inhibited by the addition of 5 μM SB431542, an inhibitor of the TGFβ signalling pathway^17^. As shown in Fig. 2b, active Smad 2/3 pathway could be inhibited upon SB431542 addition during the first 4 days of hiPSC-BAP differentiation. Then, a dramatically increase in *FABP4*, *PLIN1* and *UCP1* expression was observed (Fig. 2c). Transient inhibition of the TGFβ pathway, during the first 3 days of differentiation only, was sufficient to promote differentiation (Fig. S3). Altogether, these data underline the critical role of TGFβ pathway in switching off hiPSC-BAP differentiation.

### Identification of extrinsic factors promoting hiPSCs-BAP differentiation

**Figure 3:**
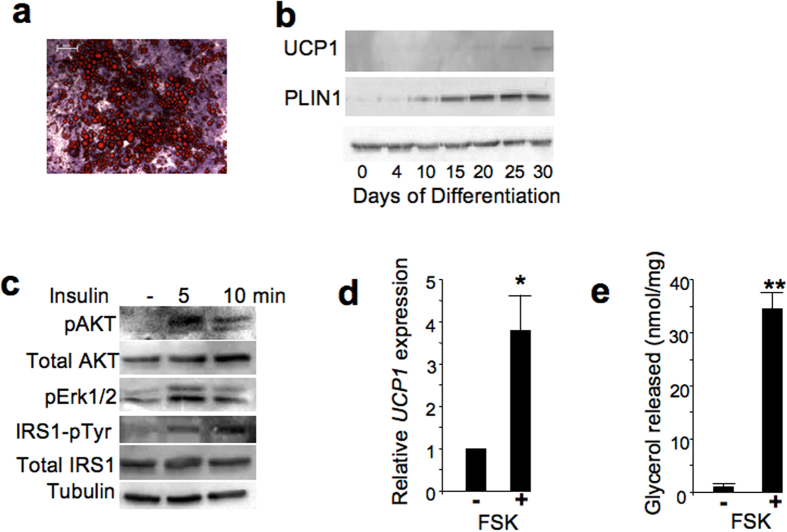
Differentiation of hiPSC-BAPs in optimized conditions. (**a**) hiPSCs-BAPs were induced to differentiate in the defined medium for 25 days. Then cells were fixed and stained with Oil Red O for lipid droplets. Bar scale: 50 μm. (**b**) hiPSC-BAPs were induced to differentiate in the defined medium and expression of UCP1 and PLIN1 at different time after adipogenic induction was analyzed by Western-blot. (**c**) Stimulation of insulin pathway : hiPSCs-BAPs were induced to differentiate in the defined adipogenic medium for 28 days as described in the Materials and Methods and then stimulated with 100 nM insulin for 5 or 15 min. Proteins were prepared and analyzed for the indicated markers. (**d**) Stimulation of *UCP1* expression by forskolin. hiPSCs-BAPs were induced to differentiate in the defined adipogenic medium for 25 days, then stimulated with 10 μM forskolin for 4 h. Values are means ± SEM. n = 4. E: Lipolysis induced by forskolin: hiPSCs-BAPs were induced to differentiate for 28 days, then stimulated with 10 μM forskolin for 5 h. *means p < 0.05 and **means p < 0.01.

**Figure 4:**
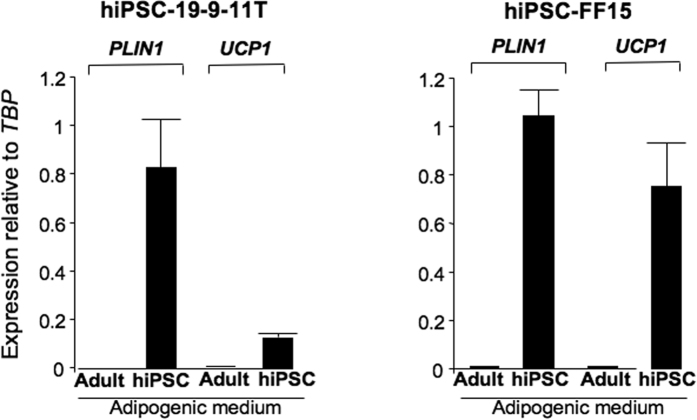
The defined hiPSC-BAP adipogenic medium supported differentiation of mesenchymal cells derived from different hiPSCs. Mesenchymal cells derived from hiPSC-19-9-11T and from hiPSC-FF15 were maintained in the traditional adipogenic medium for adult APs (Adult) or in the defined hiPSC-BAP adipogenic medium (hiPSC) for 25 days. RNAs were prepared and analyzed for expression of adipocyte markers. Values are means ± SEM. n = 4.

The commercial EGM medium contains IGF1, FGF2, VEGF, EGF, hydrocortisone and ascorbic acid with no information regarding their concentrations. We showed that FGF2, VEGF and IGF1 were dispensable for hiPSC-BAP differentiation. In contrast, hydrocortisone, ascorbic acid and EGF were required (Fig. S4A). Finally, traditional adipogenic factors supplemented with SB431542 (5 μM) and defined concentrations of ascorbic acid (25.5 μg/ml), hydrocortisone (4 μg/ml) and EGF (10 ng/ml), hereafter named defined hiPSC-adipogenic medium, dramatically enhanced adipocyte formation and expression of adipogenic markers at the protein level (Fig. 3a,b). The defined hiPSC-adipogenic medium supported differentiation at a level identical to that when cells were maintained in complete EGM2 adipogenic medium (Fig. S4B). Except SB431542, none of these essential factors was able to inhibit Smad2/3 activation (Fig. S4C). Importantly, hiPSC-BAP adipocytes were then able to respond to insulin as phosphorylated forms of IRS1, AKT and Erk1/2 were upregulated upon acute insulin administration (Fig. 3c). hiPSC-BAP progenies were also able to respond to forskolin, a chemical mimicking β-adrenergic stimulation, by increasing *UCP1* gene expression and lipolysis (Fig. 3d,e). Overall, these data showed that adipocytes generated from hiPSC-BAPs were responsive to an adrenergic stimulus and displayed an active insulin signaling pathway, the hallmark of functional brown/brite adipocytes. Interestingly, mesenchymal cells originated from two other hiPSC sources and derived by different approaches (see Materials and Methods) were not able to undergo adipocyte differentiation when maintained in a traditional adipogenic medium, as expected. However, their differentiation was dramatically promoted when maintained in the defined hiPSC-adipogenic medium (Fig. 4). Altogether, these data indicated that the set of factors identified was able to switch on differentiation of adipose progenitors derived from different human iPSC sources.

### Molecular differentiation of hiPSCs-BAPs and comparison with adipose progenitors derived from human adult adipose tissues

**Figure 5:**
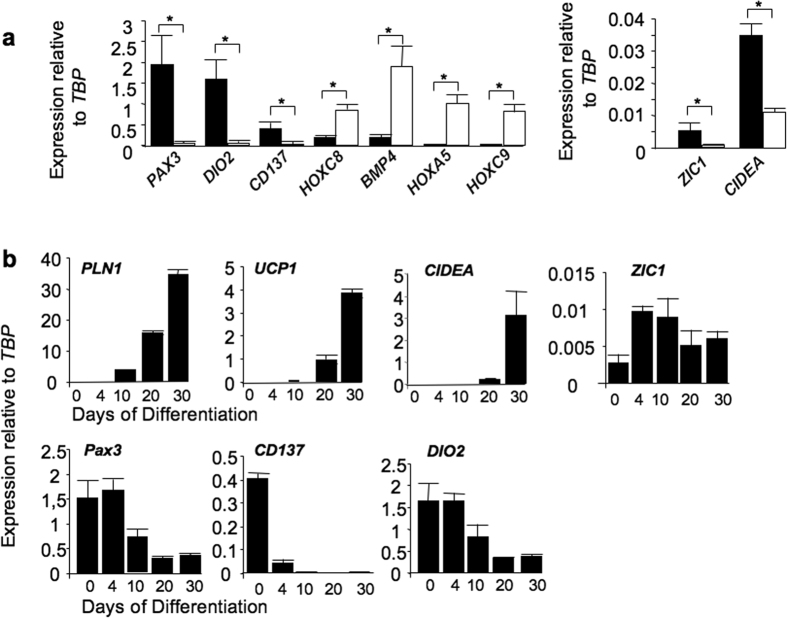
Marker gene expression in hiPSC-BAPs and -WAPs. (**a**) Expression of indicated markers was analyzed in undifferentiated BAPs (black bars) and WAPs (white bars). (**b**) hiPSC-BAPs were induced to differentiate in the defined medium and kinetics of expression of indicated markers was analyzed. Values are means ± SEM. n = 4. *means p < 0.05.

**Figure 6:**
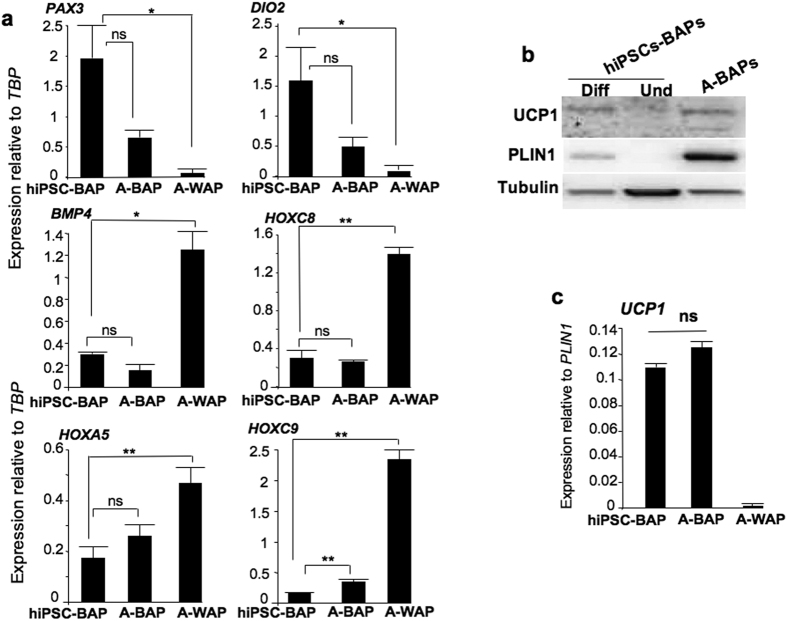
Comparison of gene expression between hiPSC-BAPs and adult-BAPs. (**a**) Expression of indicated markers was analyzed in undifferentiated hiPSC-BAPs, adult-BAPs, (A-BAPs) adult-WAPS (A-WAPS). Values are means ± SEM. n = 5. (**b)** hiPSC-BAPs and adult BAPs were differentiated and UCP1 and PLIN1 levels were analyzed by Western-blotting. All protein depots were analyzed on the same gel. (**c**) Expression of *UCP1* normalized to *PLIN1* was compared between hiPSC-BAPs, adult-BAPs (A-BAPs) and adult-WAPs (A-WAPs). Values are means ± SEM. n = 4. *means p < 0.05 and **means p < 0.01.

A number of earlier studies have proposed several specific markers for brown, brite and white adipocyte lineages in mouse. Not all of these markers are relevant in humans, but the following ones have been reported in the literature as informative^1^,^13^,^18^,^19^,^20^,^21^ *ZIC1* (brown), *PAX3, DIO2, CIDEA* (brown/brite), *CD137, TMEM26, TBX1* (brite)*, HOXC8, BMP4, HOXA5, HOXC9, TCF21* (white). Expression of these markers was investigated both in hiPSC-BAPs and -WAPs. *TMEM26, TBX1* and *TCF21* were detected but with no differential expression between both cell types (not shown). In contrast, *PAX3*, *DIO2*, *CD137*, *CIDEA* and *ZIC1* were more expressed in BAPs than in WAPs (Fig. 5a). *HOXC8, BMP4, HOXA5* and *HOXC9* were weakly, if any, expressed in hiPSC-BAPs. Altogether these data indicate that hiPSC-BAPs expressed both classical brown and brite adipocyte markers. Their kinetics of expression were investigated during hiPSC-BAP differentiation. As shown in Fig. 5b (and see Fig. 3b) expression of *PLIN1*, *CIDEA* and *UCP1* increased when cells were maintained in adipogenic medium, whereas expression of *PAX3*, *CD137* and *DIO2* was inhibited. *ZIC1* expression unchanged during differentiation. Finally, this molecular signature and the adipogenic potential of hiPSC-BAPs were compared to those of adult-APs. For that purpose, adult-BAPs and adult-WAPs were isolated from chin and knee paired fat depots, respectively, as we previously described^22^. As shown in Fig. 6a, no significant differences were observed in the level of expression of BAP markers between hiPSC-BAPs and adult BAPs. After differentiation, similar levels of *UCP1* expression were displayed in hiPSC-BAPs and adult BAPs when normalized to *PLIN1* (Fig. 6b,c). Altogether, these data indicate that hiPSC-BAPs and adult-BAPs display similar adipogenic capacity range when maintained in the appropriate adipogenic cocktail.

## Discussion

Brown/brite adipose progenitors purified from hiPSC cultures have a low adipogenic capacity compared to adult-BAPs when induced to differentiate using traditional adipogenic media. This bottleneck hampers the use of hiPSC-BAPs both in cell-based therapy and basic research. Here we show that TGFβ pathway was activated during hiPSC-BAP differentiation, likely thanks to the expression of TGFβ1 and of activin A, two potent anti-adipogenic factors. Interestingly, treatment with activin/TGFβ inhibitor SB431542 during the first days of differentiation only switched on the differentiation process. These data unlighted the critical role of TGFβ pathway and reveal a molecular mechanism regulating hiPSC-BAP adipogenesis. We observed that hiPSCs could generate a high number of adipocyte colonies when BAPs were not purified but when BAPs were maintained in the iPSC environment. This strongly suggests that non-adipose cells present in hiPSC differentiated cultures could secrete factors that are required to prime BAPs towards the adipogenic lineage. The identification of non-adipogenic cells and secreted factors supporting brown adipogenesis could be of a great interest for characterizing the brown adipocyte niche during development in humans. Factors identified in this report (EGF, ascorbic acid, TGFβ pathway inhibitor) likely belong to the niche. The low hiPSC-BAP adipogenic capacity compared to adult-BAPs is reminiscent of an observation reported by Han and colleagues^23^. These authors observed that epididymal adipose tissue, which undergoes early development in mouse, is composed of progenitor cells that lack their adipogenic capacity once isolated from the tissue. In contrast to cells derived from other fat pads that developed later, epididymal fat cells required a different micro-environment to undergo differentiation. Altogether these results underline that the micro-environment differs for adult and embryonic-like cells and suggest that distinct regulatory mechanisms regulate differentiation of embryonic-like versus adult adipose progenitors as it has been recently reported in rodent^24^.

Pathways mediating hiPSC-BAP adipogenic effects of hydrocortiosne, ascorbic acid and EGF remains to be identified. Each of these factors appeared to be required to switch hiPSC-BAP differentiation on but none of them was sufficient to drive differentiation when added individually. We have previously demonstrated the existence of an autocrine/paracrine loop comprising the TGFβ/Smad2 pathway that plays a critical negative role in adipogenesis of human adult APs^25^. This negative loop is inhibited by dexamethasone contained in the traditional adipogenic cocktail used for the differentiation of adult-APs^15^. In contrast, neither dexamethasone nor hydrocortisone was able to switch off activated Smad2 in hiPSC-BAPs and treatment with the TGFβ pathway inhibitor SB431542 was required to unlock hiPSCs-BAP differentiation. Dexamethasone is similar to the natural glucocorticoid hydrocortisone. However, dexamethasone included in the basal adipogenic medium could not replace hydrocortisone and vice versa. The molecular reasons of why both glucocorticoids were required for hiPSC-BAP differentiation remain to be investigated. Ascorbic acid was previously shown to enhance mouse adipose progenitor differentiation, and recent studies attribute its effects to the regulation of type V and type VI collagens^26^. The essential role of collagens for adipogenesis was described several years ago^27^ but one cannot rule out a metabolic effect of ascorbic acid on UCP1 expression^28^. The requirement of ascorbic acid for UCP1 expression would deserve further investigation. EGF is known to stimulate adult-BAP proliferation while maintaining their adipogenic differentiation potential^12^,^29^. However, its role in human brown adipocyte differentiation is not known. The stimulation of EGR1 expression upon EGF addition is of interest (see Fig. S4C). EGR1 has been shown to play a negative role in adipogenesis of murine 3T3-L1 cell line^30^. However, more recently it has been reported that EGR1 facilitates fat accumulation in WAT and that EGR1 null WAT express BAT markers^31^. The positive or negative role of EGR1 in EGF-induced hiPSC-BAP differentiation remains to be determined. In conclusion, these data established a defined set of factors that can induce differentiation of human BAPs derived from pluripotent stem cells at a high rate with no gene transfer. The findings in the present study may constitute a platform for *in vitro* studies and for the screening of drugs against obesity and related metabolic disorders focused on the generation of human white and brown APs. Finally, hiPSC-BAP could undergo differentiation at a level similar to adult-BAPs. This also offers a unique opportunity to investigate in animal models the therapeutic potential of hiPSC-BAPs derived from obese patients, which could lead to the development of autologous transplantation procedures to treat obesity and associated metabolic disorders.

## Methods

### Derivation of adipose progenitors from hiPSCs and from human adult stromal vascular fraction

BAPs and WAPs have been derived from the hiPSC line NOK6 as previously described^13^. Briefly, hiPSC differentiation was initiated by floating cultivation to form embryoid bodies (EBs). Retinoic acid (10^−6^M) was added or not from days 3 to 5 to generate white adipose progenitor (WAPs) or brown-like adipose progenitor (BAPs), respectively. Ten days after EB formation, EB outgrowths, that contain WAPs or BAPs at that stage, were maintained for 1 week in mesenchymal cell growth medium composed of DMEM supplemented with 10% FCS and with 5 ng/ml FGF2. BAPs and WAPs were then trypsinized and passaged with a 1:3 split ratio until achieving homogeneous CD73 labelling. A schematic diagram to explain the generation and differentiation of hiPSC-BAPs is shown in Fig. S1. Mesoderm-mesenchymal progenitors were derived from the hiPSC line 19-9-11T as described in ref. 32. The hiPSC-FF15 cell line was derived and characterized from human adult foreskin fibroblasts (HFF, Millipore) using the CytoTune®-iPS Sendai Reprogramming Kit (Thermo Fisher Scientifc), as previously described^33^. Mesenchymal Stem Cells (MSCs) were obtained after direct differentiation of hiPSC-FF15 in KO-DMEM 10% FBS with Ascorbic Acid-2-P (1 mM) and FGF2 (10 ng/mL) medium for 6 weeks on poly-D-lysine/fibronectin coated plates; cells were trypsinized every week at 1:5 dilution. MSCs were characterized by CD105+/CD73+/CD90+/CD34−/CD45−) FACS analysis. Cell cultures were more than 90% double positive after 6 passages. Adult BAPs and WAPs were derived from the stromal vascular fractions of chin and knee paired fat depots, respectively, of Caucasians women who underwent elective liposuction procedures, after obtaining their with informed consent^22^. Eleven paired chin and knee fat depots was analyzed and a representative paired of BAPs and WAPs was used to compare with hiPSC-APs.

### Optimization of hiPSC-BAP differentiation medium

hiPSC-BAPs were plated at 0.5 × 10^4^ cells/cm^2^ and maintained in proliferation in DMD low glucose medium supplemented with 10% FCS. Then, when cells reached confluence the proliferation medium was changed to differentiation medium composed of EBM-2 (Lonza) supplemented with 0.1% FCS, EGM-2 cocktail (Lonza), IBMX (0.5 mM), dexamethasone (0.25 μM), T3 (0.2 nM), insulin (170 nM) and rosiglitazone (1 μM). IBMX and dexamethasone were maintained only for the first 3 days of differentiation. This medium will hereafter be EGM2 adipogenic medium. Because the concentrations of factors contained in EGM-2 were not known, a defined medium was also established. This defined hiPSC- adipogenic medium was composed of EBM-2 (Lonza) supplemented with FCS (0.1%), SB431542 (5 μM), ascorbic acid (25.5 μg/ml), hydrocortisone (4 μg/ml), EGF (10 ng/ml), T3 (0.2 nM), insulin (170 nM), rosiglitazone (1 μM), IBMX (0.5 mM), dexamethasone (0.25 μM). IBMX and dexamethasone were maintained for the first 3 days only. The medium was changed once a week. A high differentiation rate was obtained 20–30 days after induction as between 60–80% of hiPSC-BAPs underwent adipocyte differentiation.

### RNA preparation and real-time PCR analysis

RNAs were purified on RNeasy Plus Universal columns (Qiagen, France) according to the manufacturer’s instructions. Total RNA sample concentrations were determined using a Nanodrop spectrophotometer (Thermo Scientific, Waltham, MA, USA). 1 μg RNA was used for reverse transcription. Real-time PCR assays were run on a OneStep real-time PCR machine (Applied Life Sciences) and sybergreen was from Applied Biosystems.

Normalization was performed using the geometric averages of the housekeeping gene *TBP*. Delta delta Ct values were used to achieve relative quantification of gene expression when “expression relative to *TBP”* is indicated. The sequences of primers used are in Table S2.

### Acute insulin treatment and lipolysis

hiPSC-BAPs cells were induced to differentiate in the defined adipogenic medium for 10 days. Then insulin (170 nM) was replaced by a lower concentration (0.5 nM) until day 28. Cells were maintained in DMEM supplemented with 0.2% BSA for 48 h before stimulation with 100 nM insulin for 5 or 15 min. Glycerol release into cell culture medium was determined as an index of lipolysis using Free Glycerol Reagent (Sigma Aldrich, St. Louis, MO) according to manufacturer instructions. hiPSC-BAPs were induced to differentiate in the defined hiPSC- adipogenic medium for 28 days, then maintained for 3 days in EBM basal medium supplemented with 0.2%BSA. Lipolysis was stimulated with 10 μM Forskolin for 5 hours.

### Preparation of cell extracts and Western blot analysis

Cells were rinsed with PBS and solubilized in stop buffer containing 50 mM Hepes, pH 7.2, 150 mM NaCl, 10 mM EDTA, 10 mM Na_4_P_2_O_7_, 2 mM Na_3_VO_4_, and 1% Triton X-100 supplemented with Protease Inhibitor Cocktail (Roche). The primary antibodies and dilutions used were: UCP1 (1/500, Calbiochem), Perilipin 1 (1/1000, Acris Antibodies); Tubulin (1/10000, Sigma); AKT (1/1000, Cell Signaling); Phospho-AKT (Thr308) (1/1000, Cell Signaling); Phospho-Erk1/2 (Thr202/Tyr204) (1/1000, Cell Signaling); Phosphotyrosin, clone 4G10 (1/1000, Millipore); IRS1 (1/1000, Millipore); Phospho-Smad2 (1/1000, Cell Signaling); Smad 2/3 ((1/1000, Cell Signaling). Secondary horseradish peroxidase-conjugated antibodies were purchased from Promega.

### Statistics analysis

InStat3 software and a nonparametric unpaired test (Mann-Whitney) was used for statistical analysis of the real-time PCR data. Probability values < 0.05 were considered as statistically significant and are marked with a single asterisk or with by double asterisks when p < 0.01.

## Additional Information

**How to cite this article**: Hafner, A.-L. *et al*. Brown-like adipose progenitors derived from human induced pluripotent stem cells: Identification of critical pathways governing their adipogenic capacity. *Sci. Rep.* **6**, 32490; doi: 10.1038/srep32490 (2016).

## Supplementary Material

Supplementary Information
