# Supplementary material for: Brown-like adipose progenitors derived from human induced pluripotent stem cells: Identification of critical pathways governing their adipogenic capacity
Source: Sci Rep. 2016 Aug 31;6:32490. doi: 10.1038/srep32490 (PMC5006163; doi:10.1038/srep32490)
Supplement: Supplementary Information [file srep32490-s1.pdf]

Brown-like adipose progenitors derived from human induced pluripotent stem cells: Identification of critical pathways governing their adipogenic capacity

Anne-Laure Hafner[1], Julian Contet[1], Christophe Ravaud[1], Xi Yao[1], Phi Villageois[1], Kran Suknuntha[2], Karima Annab[3], Pascal Peraldi[1], Bernard Binetruy[3], Igor I Slukvin[2], Annie Ladoux[1] and Christian Dani[1]

Supplementary information

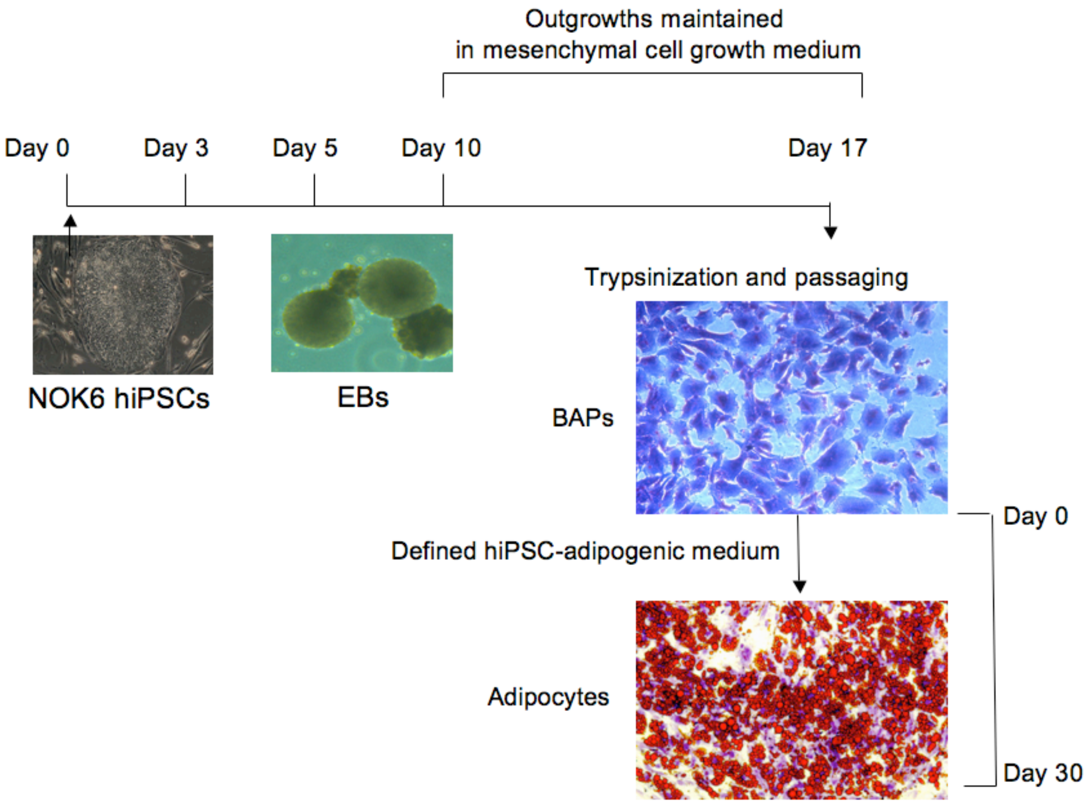

Fig.S1: Schematic diagram for hiPSC-BAP generation and differentiation

Undifferentiated BAPs were stained with Cristal Violet and adipocyte progenies with Oil red O for lipid droplets.

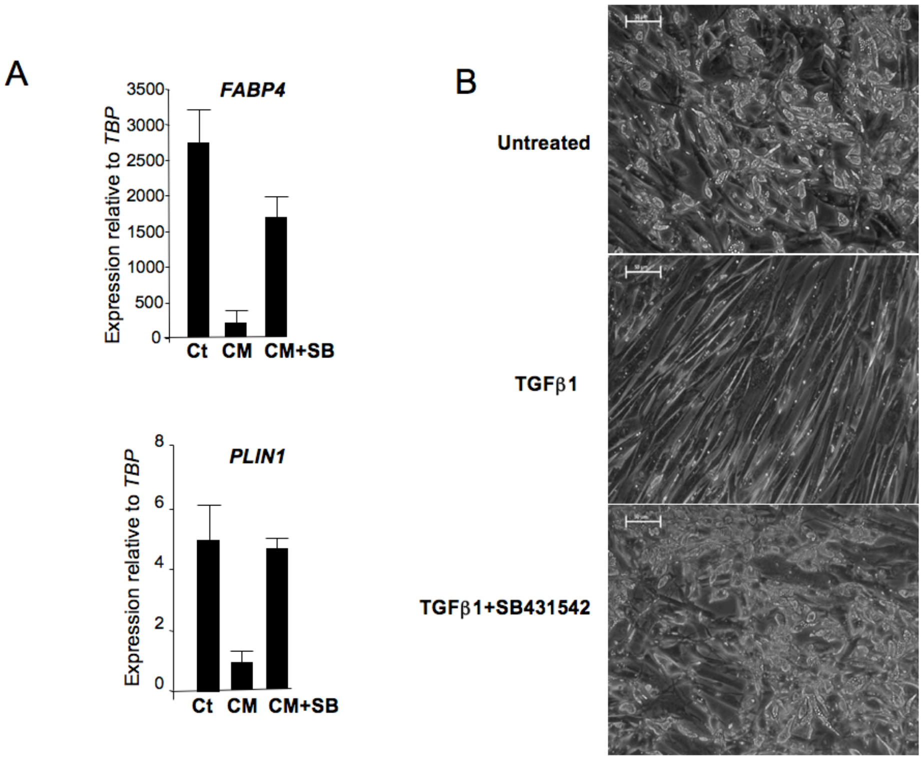

Fig S2: hiPSC-BAPs secreted anti-adipogenic factors

A) Adult BAPs derived from human chin adipose tissue were induced to differentiate in the absence (Ct) or presence of 50% medium conditioned by hiPSC-BAPs (CM), or in the presence of 50% of medium conditioned by hiPSC-BAPs supplemented with 5 μM SB431542 (CM+SB). Ten days later, RNAs were prepared and analyzed for indicated genes. Values are the average of 3 independent experiments. **B**) Microphotographs of adult-BAPs treated or not with 2 ng/ml TGFβ1 and 5 μM SB431542 during differentiation.

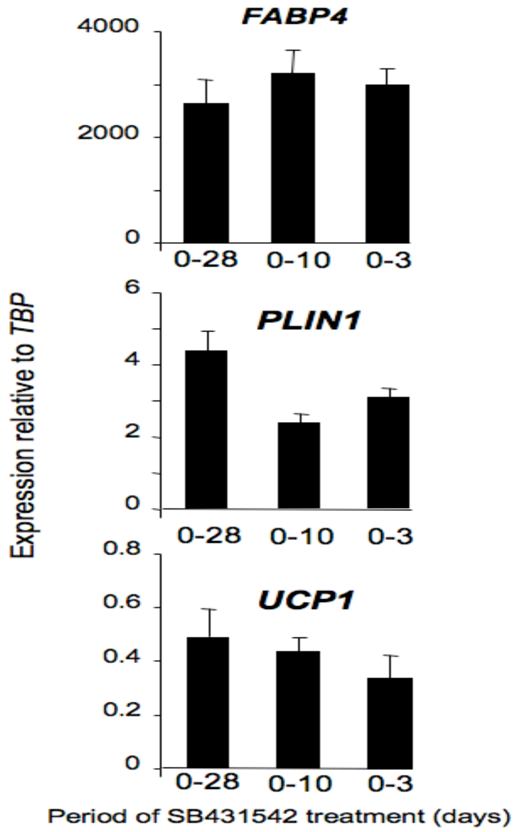

Figure S3: SB431542 treatment during different periods of time

hiPSC-BAPs were maintained for 28 days in EGM-2 adipogenic medium supplemented with 5 µM SB431542 for indicated periods of time. RNAs were prepared and analyzed for expression of indicated genes. Values are the average of 3 independent experiments. No significant statistical difference was observed.

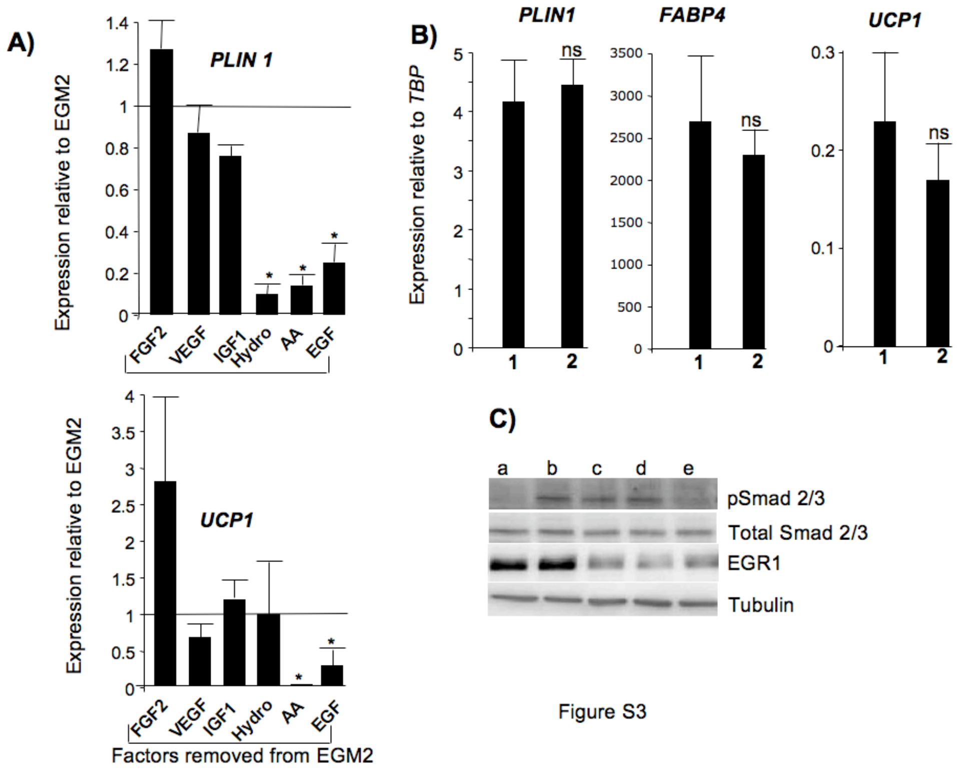

Figure S4: A) Requirement of hydrocortisone, ascorbic acid and EGF for hiPSC-BAP differentiation. hiPSC-BAPs were induced to undergo differentiation in the EGM2 adipogenic medium in which individual EGM2 factors were omitted. Twenty-five days later, RNAs were prepared and analyzed for the indicated genes. *PLIN1* and *UCP1* levels were taken as 1 in the complete EGM2 adipogenic medium. **B) hiPSC-BAP differentiation in EGM2 and defined adipogenic medium.** hiPSC-BAPs were maintained in EGM2 adipogenic medium supplemented with SB431542 (1) or in adipogenic medium supplemented with SB431542, ascorbic acid (25.5 μg/ml), hydrocortisone (4 μg/ml) and EGF (10 ng/ml). Twenty-five days later RNAs were prepared and analyzed for indicated makers. Values are means ± SEM. n=6. **C) Effect of individual compound on TGFb pathway inhibition**. hiPSC-BAPs were stimulated for 1h with indicated factors. Then proteins were prepared and analyzed for activated Smad2/3 and EGR1 expression. a) ascorbic acid (25.5 μg/ml) plus hydrocortisone (4 μg/ml) plus EGF (10 ng/ml) plus SB431542 (5μM); b) EGF (10 ng/ml); c)

hydrocortisone (4 μg/ml); d) ascorbic acid (25.5 μg/ml); e) SB431542 (5μM). * means $p<0.05$.

Basal Medium	Serum or Serum replacement	Adipogenic Factors
DMEM/F12	No	Dexamethasone (1 μM or 0.5 μM)<br>IBMX (0.5 mM or 0.25 mM)<br>insulin (170 nM)<br>T3 (0.2 nM)<br>Rosiglitazone (1 μM)
DMEM Low glucose	10% FCS	Dexamethasone (1 μM or 0.5 μM)<br>IBMX (0.5 mM or 0.25 mM)<br>insulin (170 nM)<br>T3 (0.2 nM)<br>Rosiglitazone (1 μM)
DMEM Low glucose	10% KSR	Dexamethasone (1 μM or 0.5 μM)<br>IBMX (0.5 mM or 0.25 mM)<br>insulin (170 nM)<br>T3 (0.2 nM)<br>Rosiglitazone (1 μM)
EBM-2	0.1% FCS	Dexamethasone (1 μM or 0.5 μM)<br>IBMX (0.5 mM or 0.25 mM)<br>insulin (170 nM)<br>T3 (0.2 nM)<br>Rosiglitazone (1 μM)
PBM-2	10 % FCS	PDM-2 adipogenic supplement

Table S1: **Composition of different adipogenic media used**

hiPSCs-BAPs were maintained in several culture media (named Adult-Adipogenic Media, see Materials and Methods). Media were composed of different basal media (DMEM, DMEM/F12, EBM2 or PBM) supplemented or not with fetal calf serum (FCS) or serum

remplacement medium (KSR) and supplemented adipogenic factors. The data indicated that these media supported differentiation of adult-APs at high efficiency, as previously shown, but were not able to promote hiPSCs-BAP differentiation (not shown).

Human Genes	Primers
FABP4	GGGACGTTGACCTGGACTGA<br>GGGAGAAAATTACTTGCTTGCTAAA
PLIN1	ACC CCC CTG AAA AGA TTG CTT<br>GAT GGG AAC GCT GAT GCT GTT
UCP1	GTGTGCCCAACTGTGCAATG<br>CCAGGATCCAAGTCGCAAGA
TGFb1	GGAAACCCACAACGAAATCTATGA<br>GAGAGCAACACGGGTTCAGGTA
Inhba	CTCGGAGATCATCACGTTTG<br>CCTTGGAAATCTCGAAGTGC
PAX3	ACACCGTGCCGTCAGTGAGT<br>TCGCTTTCCTCTGCCTCCTT
DIO2	GTCACTGGTCAGCGTGGTTTT<br>TTCTTCACATCCCCCAATCCT
CD137	AGCTGTTACAACATAGTAGCCAC<br>TCCTGCAATGATCTTGTCCTCT
HOXC8	GTCTCCCAGCCTCATGTTTC<br>TCTGATACCGGCTGTAAGTTTGC
BMP4	CCTGTTGTGTGCCCACTGAAC<br>ATCTCAGCGGCACCCACAT
HOXA5	CCCAGATCTACCCCTGGATG<br>CAGGGTCTGGTAGCGCGTGT
HOXC9	CAGCAAGCACAAAGAGGAGA<br>CGACGGTCCCTGGTTAAATAC
TCF21	TCA ACC TGA CGT GGC CCT TT<br>GAC TCG CAC CTC CAA GGT C
TBP	ACGCCAGCTTCGGAGAGTTC<br>CAAACCGCTTGGGATTATATTCG

Table S2: Real-Time RT-PCR Primer Sequences
